# Control Strategies for Carcinogenic-Associated Helminthiases: An Integrated Overview

**DOI:** 10.3389/fcimb.2021.626672

**Published:** 2021-03-24

**Authors:** José Manuel Correia da Costa, Maria João Gouveia, Gabriel Rinaldi, Paul J. Brindley, Júlio Santos, Lúcio Lara Santos

**Affiliations:** ^1^ Centre for the Study in Animal Science (CECA/ICETA), University of Porto, Porto, Portugal; ^2^ Centre for Parasite Immunology and Biology, Department of Infectious Diseases, National Institute for Health Dr Ricardo Jorge, Porto, Portugal; ^3^ REQUIMTE, Department of Chemical Sciences, Laboratory of Bromatology and Hydrology, Faculty of Pharmacy, University of Porto, Porto, Portugal; ^4^ Wellcome Sanger Institute, Hinxton, United Kingdom; ^5^ Department of Microbiology, Immunology & Tropical Medicine, and Research Centre for Neglected Diseases of Poverty, School of Medicine & Health Sciences, George Washington University, Washington, DC, United States; ^6^ Deparment of Urology, Clínica da Sagrada Esperança, Luanda, Angola; ^7^ Experimental Pathology and Therapeutics Group, Research Center of Instituto Português de Oncologia, Porto, Portugal

**Keywords:** helminths, carcinogenesis, chemotherapy, immunotherapy, antioxidants

## Abstract

Helminthiases are extremely prevalent in the developing world. In addition, the chronic infection with some parasitic worms are classified as carcinogenic. Therefore, it is utmost importance to understand the parasite-host interactions, the mechanisms underlay carcinogenesis and how they could be counteracted. This knowledge may ultimately guide novel control strategies that include chemotherapy-based approaches targeting these pathogens and associated pathologies caused by their infections. Little is known on how some helminthiases are associated with cancer; however, it has been hypothesized that chemical carcinogenesis may be involved in the process. Here, we summarize the current knowledge on chemical carcinogenesis associated with helminthiases, along with available therapeutic options and potential therapeutic alternatives including chemotherapy and/or immunotherapy. Ideally, the treatment of the carcinogenic helminthiases should target both the parasite and associated pathologies. The success of any chemotherapeutic regimen often depends on the host immune response during the infection and nutritional status among other factors. The close association between chemotherapy and cell-mediated immunity suggests that a dual therapeutic approach would be advantageous. In addition, there is a pressing need for complementary drugs that antagonize the carcinogenesis process associated with the helminth infections.

## Helminth Infections and Cancer

More than 10% of all cancers in the developing world are believed to be associated with infections (IARC, 2012). Whereas the infection with viruses as human papilloma virus (HPV), hepatitis C and D virus (HCV, HDV) (Ji et al., 2012; Yi and Yuan, 2017) and some bacteria are well-established biological carcinogens, helminthiases associated with malignancy remain largely unexplored (Blattner, 1999; Parkin, 2006; Brindley et al., 2015; Mentis et al., 2019). Infections with the blood fluke, *Schistosoma haematobium* (*S. haematobium*) and the Asian liver flukes, *Opisthorchis viverrini* (*O. viverrini*) and *Clonorchis sinensis* (*C. sinensis*) have been classified as Group 1 biological carcinogens: definitive causes of cancer according to International Agency for Research in Cancer (IARC), (IARC, 2012). Some close relatives of these parasites, e.g. *Schistosoma japonicum* (Group 2b) and *Opisthorchis felineus* (Group 3) are not classified as definitive biological carcinogens (Vennervald and Polman, 2009; IARC, 2012). Yet, recent findings have indicated that infection with the European liver fluke *O. felineus* may eventually lead to cholangiocarcinoma (CCA) (Gouveia et al., 2017; Pakharukova et al., 2019; Fedorova et al., 2020) and that infection with *S. japonicum* may be a risk factor for colorectal cancer (Wu et al., 2020). Curiously, chronic infections with related trematodes, the blood flukes *Schistosoma mansoni* and the liver fluke *Fasciola hepatica* have not been classified as biological carcinogens. These observations prompt questions related to the mechanisms underlying carcinogenesis during the helminth infection: how might these infections trigger cancer? (Brindley et al., 2015). Infections with parasites are recognized as both biological and chemical insults to host tissues (chemical carcinogenesis promoters) leading to inflammation, fibrosis, and changes in tissue microenvironment (Brindley and Loukas, 2017; Gouveia et al., 2017).

## Chemical Carcinogenesis May Be Responsible for Helminth Induced Malignancy

Chemical carcinogenesis (ChC), as Experimental Science, started in 1915 by Yamagiwa and Ichikawa who reproduced the carcinogenicity of coal tar in rabbit skin (Yamagiwa and Ishikawa, 1917). Later on, Elizabeth C. Miller and James A. Miller showed that ChC may occur through direct interaction of electrophilic compounds with the DNA (Miller and Miller, 1981). Several environmental factors, including physical (e.g., ionizing radiation), biological (e.g., viral pathogens), and chemicals underly the development of several human cancers (Minamoto et al., 1999). Recently, Ercole L. Cavalieri, Eleanor G. Rogan and collaborators hypothesized that catechol estrogen-3,4-quinones (CEQ) can initiate cell transformation by reacting with DNA. The production of DNA adducts creates apurinic sites in the DNA (Cavalieri et al., 1994; Cavalieri et al., 2006; Cavalieri and Rogan, 2011) and/or oxidizes the DNA. If this metabolism becomes unbalanced and generates excessive CEQ, the formation of CEQ-DNA adducts would consequently increase (Cavalieri et al., 1994; Cavalieri and Rogan, 2011). We have previously identified estrogen-like metabolites in both sera and urine from *S. haematobium*-infected patients, and parasite extracts (Botelho et al., 2013; Gouveia et al., 2015). Similarly, oxysterols were identified in *O. viverrini* and *O. felineus* (Vale et al., 2013; Gouveia et al., 2017). In both cases, tentative products derived from the interaction between parasitic-derived metabolites (e.g. estrogen- and oxysterol-like metabolites) and host DNA were identified, i.e. DNA-adducts in patients with urogenital schistosomiasis and rodent model of opisthorchiasis. These findings suggest that chemical-mediated carcinogenesis processes may underlie, at least partially, the helminthiases-associated malignancies. They also support the hypothesis that reactive oxysterol-like metabolites and oestrogen-like precursors of presumably parasite origin may be genotoxic to the host genome (Correia da Costa et al., 2014; Gouveia et al., 2015; Santos et al., 2015; Gouveia et al., 2017).

In addition to oestrogen-derived metabolites acting as chemical toxins, recent findings suggest estrogen receptors may also be involved in urogenital schistosomiasis (UGS)-associated carcinogenesis. Differential gene expression of the estrogen receptor α (ERα) is evident between UGS-associated bladder cancer and non-UGS-associated bladder cancer (Bernardo et al., 2020). There is a direct correlation between the ERα expression levels, tumor proliferation and expression of p53. The expression of ERα has been also related with the presence of parasitic eggs in bladder. On the other hand, ERβ is widely expressed in both non-UGS- and UGS-associated bladder cancer. Remarkably, these findings were supported by proteomic studies on *S. haematobium* parasites and, moreover, the *in vitro* activation of ERα promotes cell proliferation in ERα-expressing bladder cancer cells (Bernardo et al., 2020).

**Figure 1:**
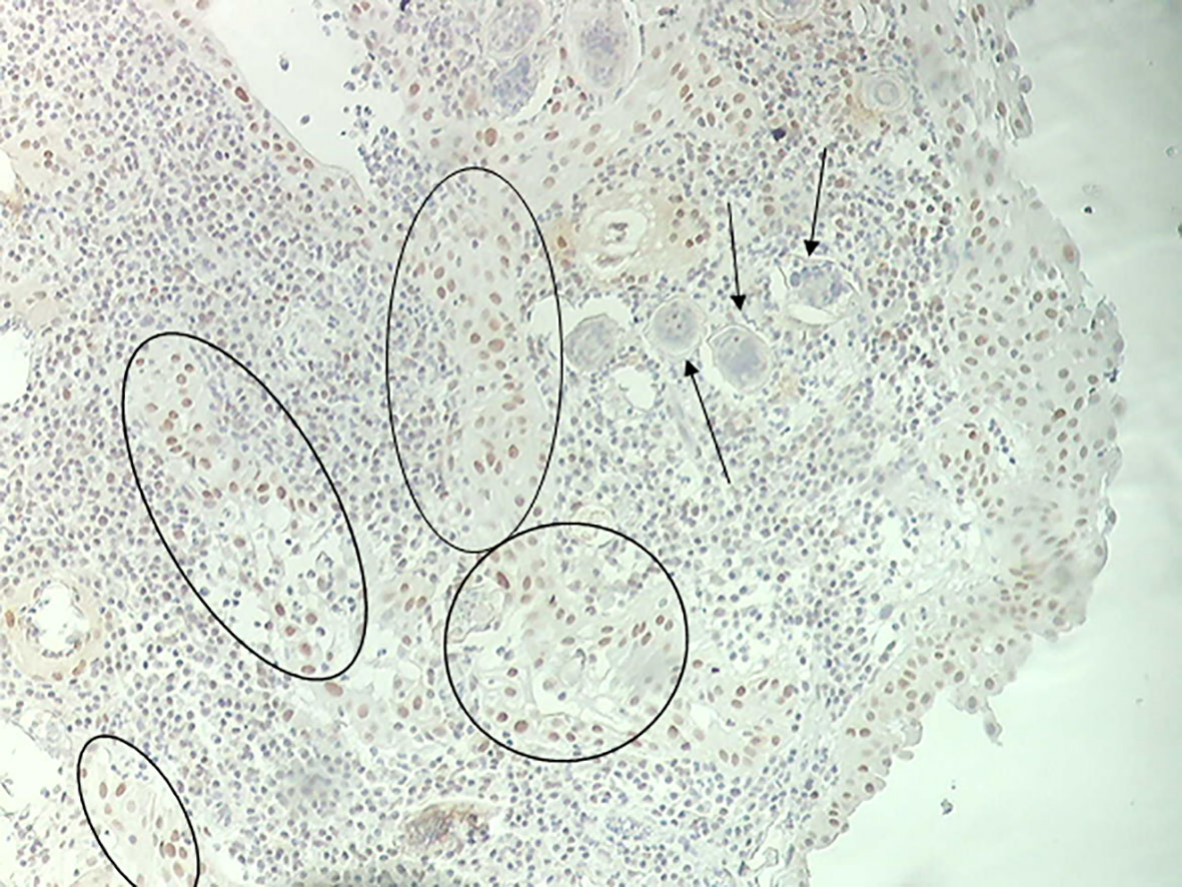
Immunohistochemistry of bladder mucosa evidencing p53 tissue deposition (black circles) and *S. haematobium* eggs (black arrows).

**Figure 2:**
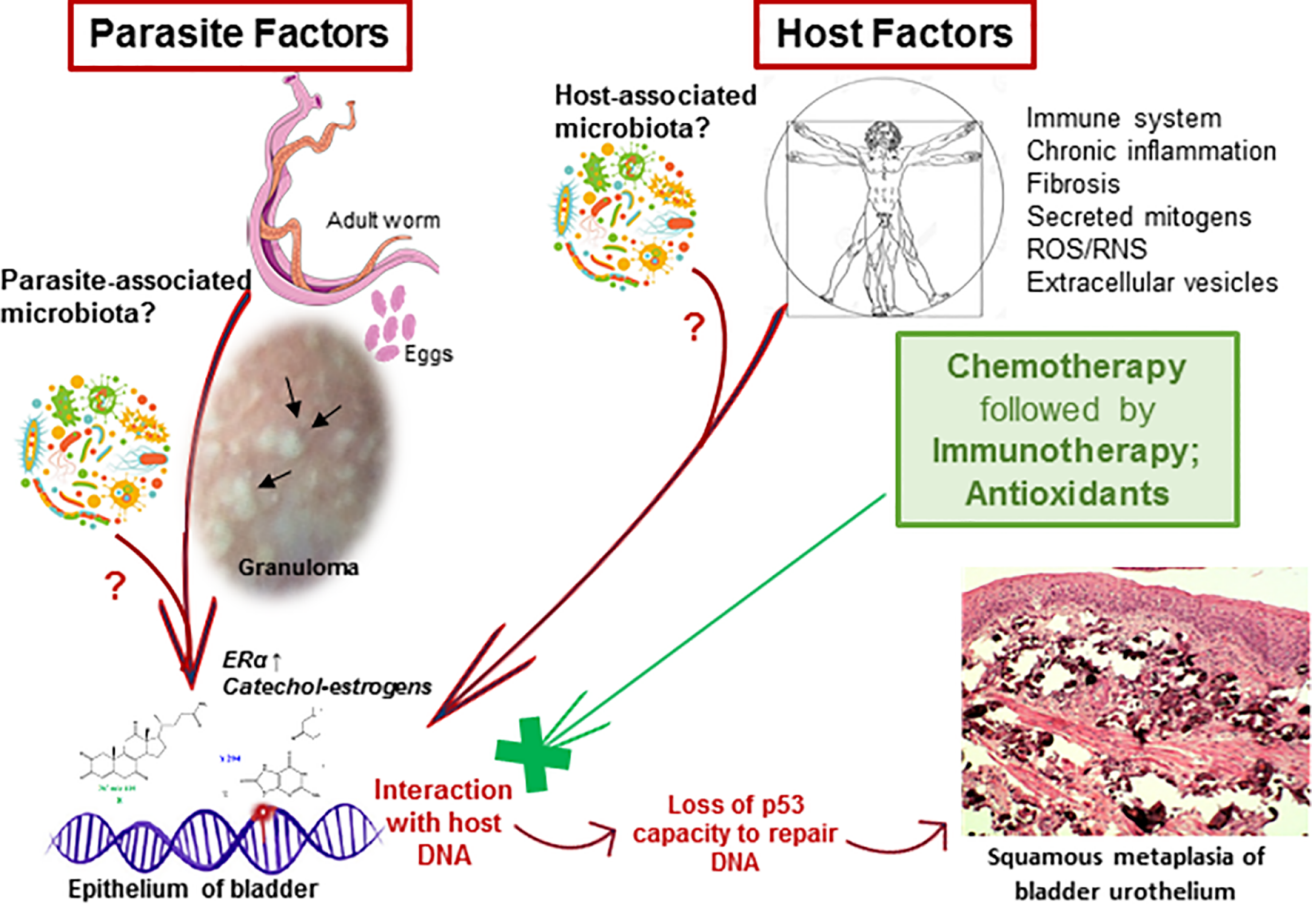
Schematic depiction of hypothesized carcinogenesis induced by urogenital schistosomiasis (UGS). Several risk factors may play a role in the onset of UGS-associated bladder cancer. Both parasite- and host-associated factors may be involved during carcinogenesis. Chronic inflammation, fibrosis and granulomas (black arrows) induced by the eggs of the parasite may have a role in carcinogenesis. Also, mitogens and reactive metabolites of schistosome origin (e.g., catechol estrogens) could interact with host DNA to form adducts and to induce apurinic sites. These could eventually lead to p53 mutations that consequently lost the ability to repair DNA. Therefore, the mutations may accumulate and ultimately underlie the development of UGS-associated squamous cell carcinoma. Co-factors, such as a tentative microbiota associated with the parasite (Formenti et al., 2020), host microbiota dysbiosis during the infection, and other host-associated factors such as the formation of ROS/RNS during chronic inflammation, and fibrosis, tobacco smoke, gender, age and impaired immune system, are known to influence the development of bladder cancer. Laboratory studies have suggested that the administration of antioxidants may counteract the interaction of reactive metabolites with host DNA, tentatively, preventing the development of pre- and carcinogenic lesions and may contribute to the amelioration of the pathogenesis associated to the UGS.

We hypothesized that the interaction of estrogen-like metabolites with host DNA may have a potential role in inducing dysregulation of p53 during UGS (Vale et al., 2017). Bladder tissue from schistosomiasis patients expressed p53 (**Figure 1**) and most likely it is mutated without capacity to repair DNA (Santos et al., 2015). *S. haematobium* eggs express sialyl-Lewis, sLea and sLex antigens, in mimicry of human leukocytes glycosylation, and may play a role in cancer metastasis (Santos et al., 2015). Recently, we reported the effects of culturing human epithelial cells established from normal urothelium (HCV29) and normal cholangiocytes (H69), in the presence of *S. haematobium* or *S. mansoni* eggs. Intriguingly, the estrogen receptor and β-estradiol were predicted to be altered in urothelial cells exposed only to *S. haematobium* but not *S. mansoni* eggs. In addition, genes involved in the p53 pathway were downregulated when exposed to eggs from species of both schistosomes (Nacif-Pimenta et al., 2019). Concerning liver flukes, a comparison between the mutation profiles between opisthorchiasis-associated CCA and non-opisthorchiasis-associated CCA reveals a significant higher number of mutations in p53, along with other somatic and epigenetic lesions, in the former compared to the latter (Jusakul et al., 2015; Jusakul et al., 2017). Whereas the full complement of metabolites released by this parasite remains generally to be investigated (Brindley and Loukas, 2017), a granulin secreted by the liver fluke, termed Ov-GRN-1, has been studied in depth for several years now (Smout et al., 2015; Dastpeyman et al., 2018). This protein induces proliferation of cholangiocytes, the cell of origin of CCA. Remarkably, it was recently reported that infection of hamsters with gene-edited liver flukes, mutated by CRSPR/Cas programmed editing of the granulin gen locus, resulted in markedly reduced pathology (Arunsan et al., 2019). Besides the risk factors mentioned above, infections with schistosomes disturb the host microbiota leading to intestinal dysbiosis before oviposition (Jenkins et al., 2018). In addition, the original composition of host microbiota might affect the susceptibility to schistosome infection (Córtes et al., 2020). In the context of UGS-bladder cancer, the urinary microbiome might be relevant for biomarker discovery to advance novel diagnostics and treatment (Adebayo et al., 2017). In rural regions of Thailand and Laos where opisthorchiasis and opisthorchiasis-associated CCA are highly endemic, traditional dietary practices involved eating of raw fish, and hence repeated exposure to liver flukes, and consumption of nitrosamine-contaminated food, particularly fermented fish products, represent the major risk factors for CCA (Sripa et al., 2007; Siriraj et al., 2016). Additional risk factors that are now coming under closer scrutiny include the carriage of *Helicobacter* species and other microbiome changes within the biliary tract that influence the inflammatory milieu (Sripa et al., 2018). A new paradigm has been proposed that indicates that most cancers originate from biological or chemical stimuli followed by chronic inflammation, fibrosis, and changes in the tissue microenvironment that eventually lead to a pre-cancerous lesion (Brücher and Jamall, 2014). It is reasonably believed that that paradigm could reflect the progression of UGS and associated bladder cancer. Or more likely, knowing that cancer is a multifactorial diseases (Clavel, 2007), the risk factors noted above may act in concert during the development of SCC-associated to UGS (**Figure 2**). Accordingly, therapeutic programs against carcinogenic helminthiases should consider a dual approach: treatment against both the parasite and associated pathology, including pre-carcinogenic lesions (**Figure 2**).

## Can Chemotherapy and Immunotherapy Combined Be an Efficient Option to Treat Helminthiases and Associated Pathologies?

Currently, praziquantel (PZQ) is used to treat schistosomiasis and opisthorchiasis, and currently employed in mass drug administration (MDA) programs across endemic regions (Cioli and Pica-Mattocia, 2003; Fenwick et al., 2003; Utzinger and Keiser, 2004; Caffrey, 2007). Of concern, parasite isolates collected in the field with decreased sensitivity to PZQ have been reported (Ismail et al., 1996; Fallon, 1998; Pica-Mattocia and Cioli, 2004; Melman et al., 2009; Crellen et al., 2016). Although PZQ can eliminate the parasites, it does not reverse the pathological sequelae after the chronic infection. Altogether, the evidence points to the need for novel therapeutic approaches against parasites. In many cases, the new drugs or derivatives exhibit lower therapeutic potency (Utzinger et al., 2011). Alternative approaches, e.g., drug repurposing, combination of drugs or other active agents and immunotherapy would be promising for the treatment of these helminthiases. Ideally, their treatment would not only treat the infection, by eliminating the parasite and ameliorating associated disease, and in addition, thwart the development of malignancy.

Combinations of drugs have been used in other infectious diseases such as malaria, and tuberculosis (Kerantzas and Jacobs Jr, 2017; Mokhtari et al., 2017; Alven and Aderibigbe, 2019). Using drugs with diverse modes of actions may be advantageous and render the treatment more effective than single drug. In the context of helminth infections, using a combination of two drugs, one active against adult worm and the other against larval stages should be more effective to eliminate the parasite. Several clinical trials have been evaluated the combination of antiparasitic drugs for UGS and compared with single dose (Pugh and Teesdale, 1983; Wang et al., 2004; Keiser et al., 2010; El-Beshishi et al., 2013; Keiser et al., 2014; Gouveia et al., 2018). Most trials confirmed that a combined regimen is more effective than a single treatment leading to an elevated cure rate (Gouveia et al., 2018). Our research group reported an increase of the PZQ and artesunate (AS) efficiency against larval stages of the parasite when combined with antioxidants *N*-acetylcysteine (NAC) and resveratrol (RESV) (Gouveia et al., 2019a). The use of antioxidants either alone or combined with drugs might be valuable for therapy of helminthiasis-induced malignancy. During schistosomiasis and opisthorchiasis, alterations of cellular antioxidant systems originated during the host immunological response have been described, with the production of reactive oxygen species (ROS) (Maizels et al., 1993; Gharib et al., 1999). The protective effects of NAC and resveratrol (RESV) against host tissue fibrosis maybe due to the inhibition of genotoxic metabolites produced by the parasite, that could eventually initiate cell transformation that leads to SCC and CCA (Seif el-Din et al., 2011; Douiev et al., 2017). Indeed, *in vitro* studies demonstrated that RESV and NAC inhibited the formation of these potentially parasitic genotoxic metabolites (Gouveia et al., 2019b). Thus, antioxidants would be an attractive therapeutic option to counteract reactive xenobiotics arising from oxidation (Soliman et al., 2008; Charoensuk et al., 2011), and inflammatory responses directed at schistosome eggs (Seif el-Din et al., 2011). The combination of one drug that exerts anthelmintic activity with antioxidants might improve biochemical, pathological, and immunological parameters associated to the infection (see Gouveia et al., 2018).

Other advantages of using combinatorial therapy include the delay of drug resistance development, synergy of action and hence a tentative reduction of the therapeutic doses required (Chen and Lahav, 2016). However, there are some limitations of combinatorial therapy such as potential increase of cytotoxicity (Humpfrey et al., 2011). In the specific case of the use of antiparasitic drugs with antioxidants, this is not expected since antioxidants are considered very safe agents (Saso and Firuzi, 2014). Nonetheless, this should be evaluated in future studies.

Novel control strategies based on immunotherapy against UGS and opisthorchiasis are being tested. These involved the prevention and/or treatment of disease with drugs that stimulate the immune response (Naran et al., 2018). This could be achieved by either the exploitation of parasite-derived antigens or the administration of drugs or other active compounds. Hundreds of schistosome antigens have been studied as promising vaccine candidates. Remarkably, four vaccines (Sh28GST, Sm-14, Sm-TSP-2, and Sm-P80) are currently at differing clinical phases (reviewed in McManus, 2020). The most promising parasite-derived antigen is schistosome 28-kDa glutathione S-transferase of *S. haematobium* (Sh28GST, Bilhavax) that currently is in phase 3 of human clinical trials (Riveau et al., 2018). This candidate induces a strong mucosal immune response associated with Th2-type and regulatory IL-10 cytokines either in animal model, or in human (Riveau et al., 2012; Riveau et al., 2018). Hypothetically, one of the most effective way to control the disease may be a combined action between the use of drugs such as PZQ and vaccines. In a recent study, a simulation was performed to compared the effect of mass drug administration (MDA) alone with vaccination plus MDA against schistosomiasis. The findings indicate that vaccination accompanied with MDA would accelerate and prolong the impact by reducing the reinfection rate and the number of eggs released by residual worms (Alsallaq et al., 2017; Kura et al., 2019). In case of opisthorchiasis, a recombinant protein termed rOv-LEL-TSP-2 of the large extracellular loop of tetraspanin-2 of *O. viverrini* was evaluated in a rodent model increasing levels of several Th1 type cytokines (Phung et al., 2019) and extracellular vesicles (EVs) resulted in partial of protective efficacy against *O. viverrini* infection (Chaiyadet et al., 2019). To the best of our knowledge, vaccines for opisthorchiasis or clonorchiasis have not been evaluated in human trials yet.

The use of antioxidants have been evaluated in animal model of schistosomiasis and opisthorchiasis, either alone or in combination with antiparasitic drugs. This scheme of treatment may enhance the host immune response against the infection and, in parallel ameliorate associated pathologies (Allam, 2009; Aires et al., 2012; Wonchalee et al., 2013; Kamel and El-Shinnawy, 2015; Sheir et al., 2015).

Despite these encouraging findings further studies are needed. It is important to note that the immune response observed in animal models depends on the rodent species and strain and there is a possibility that any given response in the rodent model may not mirror the pathophysiological and immunological events in humans (Herati and Wherry, 2018). Therefore, ideally more reliable models of these diseases to perform immunologic studies are warrant. Also, the identification of key immunological targets should be pursued as well as the role of Th1 and Th2 responses and the balance between these two require further elucidation (McManus, 2020). We believe that the combination of chemotherapy and immunotherapy should be further investigated. Additionally, immunotherapy could play a valuable role in prevention or treatment of cancers induced by infections. This approach has been considered in the treatment of several other cancers (Farkona et al., 2016).

Thus far, it is unclear which components of *S. haematobium* eggs are pro-oncogenic, but it is known that eggs alone without the presence of adult worms could trigger the inflammatory response and cell proliferation (Fu et al., 2012; Nacif-Pimenta et al., 2019). Notably, Santos et al. (2015) outlined findings that provide insights on the glycosylation patterns of *S. haematobium* eggs and speculate about a possible model for the recruitment of eggs to the bladder wall. These observations led to important questions: Has the parasite evolved glycosylation patterns that mimic those of the human host? Would these insights represent informative guides to develop novel therapeutic strategies, namely glycoconjugate vaccines?

### Concluding Remarks—The Critical Need for an Integrated Approach

Combined chemotherapy alone or in association with vaccines, will develop into novel therapeutic approaches. However, it is unlikely that they will achieve sustainable medium- to long-term control for schistosomiasis and opisthorchiasis *per se*. A better understanding of socioecological context of parasite transmission and links of schistosomiasis with poverty are crucial to achieve effective control programs (Utzinger et al., 2003; Singer and Castro, 2007; Bruun and Aagaard-Hansen, 2008; Parker et al., 2008; Utzinger et al., 2009; Gray et al., 2010; Tangkawattana and Sripa, 2018). Consensually, such programs need to include education, snail control, better access to clean water and sanitation. Incorporate local resources and leadership to improve and enhance the existing health system may be critical (Utzinger et al., 2011). In this regard, China provides a good example of what can be achieved by implementing an integrated and sustainable control strategy against schistosomiasis. Combining experience among all the actors involved, using local resources for control and implementing an integrated control approach adapted to the local ecological settings will be crucial. This will ultimately lead to a reduction or elimination of the transmission of schistosomiasis and other helminthiasis, and the severe associated pathologies including cancer (Utzinger et al., 2005; Wang et al., 2008; Wang et al., 2009; Gray et al., 2010; Spiegel et al., 2010).

## Data Availability Statement

The original contributions presented in the study are included in the article, further inquiries can be directed to the corresponding author.

## Author Contributions

JC conceptualized and wrote the first draft of the manuscript. MG, PB, GR, JS, and LS reviewed and suggested alterations that were included in the manuscript. All authors contributed to the article and approved the submitted version.

## Funding

This work received financial support from PT national funds (FCT/MCTES, Fundação para a Ciência e Tecnologia and Ministério da Ciência, Tecnologia e Ensino Superior) through the project UIDB/ 00211/2020. PB acknowledges support from award RO1CA164719 from the National Cancer Institute (NCI), National Institutes of Health (NIH), USA. The content is solely the responsibility of the authors and does not necessarily represent the official views of the FCT, NCI, NIAID, or NIH.

## Conflict of Interest

The authors declare that the research was conducted in the absence of any commercial or financial relationships that could be construed as a potential conflict of interest.
